# Loving Others: The Impact of Compassionate Love on Later-Life Psychological Well-Being

**DOI:** 10.1093/geroni/igaa057.1554

**Published:** 2020-12-16

**Authors:** Eva Kahana, Tirth Bhatta, Boaz Kahana, Nirmala Lekhak

**Affiliations:** 1 Case Western Reserve University, Cleveland, Ohio, United States; 2 University of Las Vegas Nevada, Las Vegas, Nevada, United States; 3 Cleveland State University, cleveland, Ohio, United States; 4 University of Nevada, Henderson, Nevada, United States

## Abstract

Existing scholarship in social gerontology has surprisingly paid little attention to broader loving emotions, such as compassionate and altruistic love, as potentially meaningful mechanisms for improving later life psychological well-being. This study examined the influence of feeling love toward other persons and experiencing love from others on later life psychological well-being. We conducted a 3-wave longitudinal study of a representative sample of 340 ethnically heterogeneous community dwelling older residents of Miami, Florida. The increase in feeling of being loved (β=-1.53, p<0.001) and love for others (β=-1.43, p<0.001) led to decline in odds of reporting greater level of depressive symptoms over time. The odds of reporting higher level of positive affect were significantly greater for older adults who reported feeling loved by others (β=1.16, p<0.001) and expressed love for other people (β=1.18, p<0.01). Older adults who felt loved had 0.92-point lower ordered log odds of reporting higher negative affect than those who reported lower level of love. The impact of compassionate love on depressive symptoms and negative affect remained statistically significant even after adjustment for altruistic attitudes and emotional support. The influence of loving emotions on positive affect was, however, explained by altruistic attitudes and emotional support. Our findings underscore the powerful influence of both receiving and giving love for the maintenance of later life psychological well-being. We offer support for the expectation that love is a significant force in the lives of older adults that transcends intimate relationships.

